# Development of a Novel High-Temperature Microemulsion for Enhanced Oil Recovery in Tight Oil Reservoirs

**DOI:** 10.3390/ma16196613

**Published:** 2023-10-09

**Authors:** Lixiao Xiao, Jirui Hou, Weiju Wang, Infant Raj

**Affiliations:** Unconventional Petroleum Science and Technology Research Institute, China University of Petroleum (Beijing), Beijing 102249, China; xiaolx713@foxmail.com (L.X.); wangweijuyyn@163.com (W.W.)

**Keywords:** high-temperature tolerance, microemulsion, enhanced oil recovery, tight oil reservoirs, imbibition mechanisms

## Abstract

This work focuses on the development of a novel high-temperature microemulsion for enhanced oil recovery in tight oil reservoirs. Microemulsions are a type of mixture that has properties of both liquids and solids; they have shown significant potential for improving oil recovery through spontaneous imbibition. Herein, a high-temperature-tolerant lower-phase microemulsion using a microemulsion dilution method was developed. The properties and morphological characteristics of the microemulsion were evaluated and proposed a mechanism for enhanced spontaneous imbibition oil recovery using imbibition tests and CT scanning technology. The results of the study showed that the optimum concentration of the microemulsion was 0.2 wt% and that it had good thermal stability, small droplet size, lower interfacial tension, good wettability alteration ability, and minimum adsorption loss. The imbibition and CT experiments demonstrated that the reduction in oil/solid adhesion was due to the synergistic effect of IFT reduction and wettability alteration and the ability to increase the imbibition distance through a larger self-driving force. The study concludes that the solubilization coefficient and self-driving force were defined and calculated to quantitatively analyze the imbibition mechanisms and the results showed that the reduction in oil/solid adhesion was due to the synergistic effect of IFT reduction and wettability alteration and the ability to increase the imbibition distance through a larger self-driving force.

## 1. Introduction

As oil consumption continues to rise, the amount of conventional oil and gas re-serves has been significantly reduced [1,2]. Therefore, it is urgent to find new resources to alleviate the supply–demand contradiction caused by low production and high consumption of oil. The large-scale development of tight oil in north America has re-versed a 24-year decline in oil production in the United States, providing new directions to increase oil production. Recently, tight reservoirs have become a hot spot in the field of international oil exploration and development due to their rich hydrocarbon reserves [3]. Compared to high/mid-high permeability reservoirs, tight reservoirs are more difficult to develop due to the presence of micro/nano-scale pore throats (<1 μm), extremely low porosity (<10%), and unevenly low permeability (<0.1×10^−3^ μm^2^) [4]. In water-wet tight reservoirs, the process of the wetting phase fluid (water phase) replacing the non-wetting phase fluid (oil phase) is defined as spontaneous imbibition in oil reservoirs [5,6]. Due to the tiny throats, the capillary effect is particularly obvious and the capillary force becomes the main driving force during the spontaneous imbibition process [7,8]. Therefore, spontaneous imbibition has become an important approach to replacing oil and improving the imbibition effect is a good choice to enhance oil recovery (EOR) in tight oil reservoirs [9,10,11].

Polymer has a relatively high molecular weight and viscosity, making it difficult to enter tight reservoirs, resulting in poor applicability and lower oil recovery. Surfactants can change the wettability of rock surfaces from oil-wet to water-wet, reduce the oil–water interfacial tension (IFT), and emulsify crude oil. As a result, it is a commonly used agent in spontaneous imbibition that can improve oil recovery in tight reservoirs [12,13]. Junrong Liu et al. [14] investigated the ability of anionic surfactant and nonionic surfactants to enhance spontaneous imbibition oil recovery (SIOR). Experimental results indicate that surfactants could enhance SIOR by altering the shale wettability and reducing the IFT, with the anionic surfactant having the greatest SIOR of 35% mainly due to the strong wettability alteration ability. Junru Wang et al. [15] studied the effects of surfactants on SIOR in tight reservoirs using Nuclear Magnetic Resonance (NMR). Compared with water imbibition, surfactant imbibition has greater imbibition depth and a higher oil recovery rate by 17.19%.

However, the applications of surfactants in tight reservoirs are still extremely limited. Strand et al. [16] indicated that high temperature has a seriously negative impact on the stability of surfactant solutions, leading to a low SIOR. Moreover, the adsorption and retention of surfactants are serious in tight oil reservoirs, which greatly reduces the effective migration distance and oil displacement efficiency of surfactants [17,18]. Moreover, surfactants can emulsify crude oil into multiple emulsions such as W/O/W and O/W/O, making it difficult to demulsify the multiple emulsion [19,20,21]. Considering the limitation of surfactant applicability and the lower oil recovery caused by high adsorption loss during, it is urgent to find an imbibition agent with small adsorption loss and a significant spontaneous imbibition effect for high-temperature tight oil reservoirs.

Microemulsion is a highly dispersed system that is isotropic, thermodynamically stable, transparent, or translucent which is formed spontaneously by two mutually immiscible liquids under the action of surfactants and co-surfactants [22]. The droplet size of the microemulsions is generally 1–100 nm with remarkable solubilization and permeation abilities [23]. The formation and arrangement of the internal “shell-core structure” not only significantly reduces the adsorption loss of surfactants but also solubilizes crude oil in the micro-nano scale pore throats [24,25,26]. Recently, microemulsions have been widely applied in the petroleum industry, especially in EOR due to their ultra-low IFT, excellent wettability alteration effect, and lower adsorption loss [27]. With the development of unconventional reservoirs, microemulsions are generally used in low-permeability reservoirs and ultra-low permeability reservoirs to displace oil by microemulsion or in situ microemulsion [28]. However, there are few studies on its application in tight reservoirs, especially in high-temperature reservoirs. At the same time, there is no clear quantitative explanation for the microemulsions’ imbibition mechanisms.

In order to enhance the oil recovery of high-temperature tight reservoirs, in the study, we prepared a high-temperature-tolerant lower phase microemulsion (HTLP-ME) by dilution method and the imbibition mechanisms were also studied [29]. The high-temperature stability was determined by turbidity tests at different temperatures. The IFT and wettability alteration experiments of the microemulsion at different concentrations were measured and the adsorption rate and adsorption loss were investigated by a static adsorption experiment. At the same time, the self-emulsification ability of the microemulsion was discussed in the emulsifying and solubilizing experiments. Additionally, spontaneous imbibition experiments with CT scanning technology were carried out to clarify the spontaneous imbibition mechanism of the microemulsion and confirm the EOR effect in the tight cores.

## 2. Materials and Methods

### 2.1. Material and the Synthesis of HTLP-ME

#### 2.1.1. Materials

(1) Experimental reagent

The nonionic surfactant is fatty alcohol polyoxyethylene ether MOA15, AR, purchased from Shanghai Aladdin Biochemical Technology Co. The general molecular formula is 
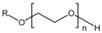
 where n is 15 and R is C12~C14, this is C12.

The co-surfactant is triethylene glycol, AR, purchased from Shanghai Aladdin Biochemical Technology Co. (Shanghai, China). The cationic surfactant SS-2306 is a quaternary ammonium salt surfactant purchased from Qingdao Changxing Technology Co., Ltd. (Qingdao, China). The oil phase used in the preparation of microemulsion is No.3 White Oil obtained after deep refining by using hydrogenation of raw materials. Its main components are light alkane compounds, such as aliphatic and aromatic hydrocarbons, and its viscosity is about 3 mPa·s. The oil phase used in the performance evaluation and imbibition experiments is kerosene, GR, purchased from Shanghai Aladdin Biochemical Technology Co. (Shanghai, China).

(2) Experimental cores

The parameters of the cores used for the imbibition experiment are shown in Table 1.

#### 2.1.2. The Synthesis of HTLP-ME

According to the mass ratios shown in Table 2, the nonionic surfactant MOA15, cationic surfactant SS-2306, oil phase No.3 white oil, co-surfactant triethylene glycol, and aqueous phase fresh water are mixed to form a mixture of 100 g.

**Figure 1:**
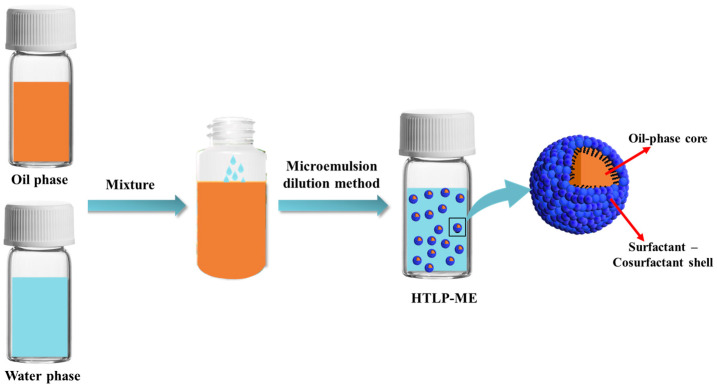
The process of preparing HTLP-ME.

The preparation process of HTLP-ME is shown in Figure 1. The mixture of oil and water phases is stirred well under lower energy conditions (50 r/min, room temperature) and it can be spontaneously emulsified into a clear and transparent microemulsion concentrate in a few minutes. Dilute the microemulsion concentrate with fresh water to prepare different concentrations of HTLP-ME. The HTLP-ME is distributed with the “shell-core structure” with the oil phase as the lipophilic core and the surfactants and co-surfactants as the hydrophilic shell.

### 2.2. Characteristics of HTLP-ME

#### 2.2.1. The Droplet Size and Morphology

The HTLP-ME with a mass concentration of 0.2 wt% was prepared. Using a microsyringe to absorb 5 μL of the tested solution, it was then added to the ultra-thin carbon film which was dried in air at room temperature. Then, the ultra-thin carbon film was transferred into the viewing chamber of the cryo-TEM to qualitatively observe the morphology and droplet size of the “shell-core structure” formed by self-emulsification in HTLP-ME. At the same time, the droplet size distribution of the “shell-core structure” was quantitatively analyzed by a nano-laser particle size and ZETA potential analyzer. The experimental results of this work, such as the droplet size, turbidity, contact angle, adsorption, and imbibition recovery, were all performed in three parallel experiments and then averaged for analysis.

#### 2.2.2. High-Temperature Resistant Experiment

The prepared 0.2 wt% HTLP-ME was divided into five equal parts to put into the total phosphorus and total nitrogen screw colorimetric tubes (50 mL). And then, the five tubes were placed into the thermostats at different temperatures: 20 °C, 45 °C, 70 °C, 95 °C, and 120 °C. After standing for two weeks, the five tubes were taken out and then the HTLP-ME were poured into five ordinary glass bottles. The appearance of HTLP-ME change with temperature can be observed.

The steps to determine the high-temperature stability of HTLP-ME by the turbidimetric method are as follows: a. Deionized water is filtered two or three times through a filtration device to prepare zero turbidity solution. b. In total, 100 mL of standard solution with a turbidity of 10 NTU is prepared. c. The zero-turbidity water is used to zero the turbidity meter. The 10 NTU standard solution is placed in the sample cell for calibration. At the end of the calibration, the turbidity meter data are kept at 10 NTU. d. The liquid to be tested is placed in the sample cell and the shown data are the turbidity of the tested liquid.

### 2.3. Interfacial Properties

#### 2.3.1. Oil–Water Interfacial Property—IFT

HTLP-ME with concentrations at 0.05 wt%, 0.1 wt%, 0.2 wt%, 0.3 wt%, 0.5 wt%, and 0.7 wt% were prepared as tested liquids. The ITF was measured by the rotating droplet interfacial tension-meter TX-500. The experimental process is as follows.

(1) The glass capillary was rinsed 2 to 3 times with distilled water and then rinsed with the tested liquid. The liquid to be tested was then injected to fill the glass capillary;
(2) Overall, 2 μL of kerosene sample pipetted with a 5 μL micro-syringe was injected into the middle of the glass capillary and then the glass capillary was placed into the sample cell of the rotating droplet interfacial tension-meter TX-500;
(3) To increase the temperature to 50 °C, the IFT between kerosene and different concentrations of HTLP-ME was measured at a rotational speed of 6000 r/min and a time interval of 1 min.

#### 2.3.2. Solid–Liquid Interfacial Property—Wettability

HTLP-ME with concentrations of 0.05 wt%, 0.1 wt%, 0.2 wt%, 0.3 wt%, 0.5 wt%, and 0.7 wt% were prepared as the liquids to be tested.

(1) Quartz flakes (2.5 cm × 2.5 cm × 0.5 cm) were soaked in 1 wt% hydrochloric acid to remove impurities and then removed to wipe and dry;
(2) The quartz flakes were aged in silicone oil with a viscosity of 100 mPa·s for a week. After that, they were cleaned with kerosene and dried to obtain oil-wet quartz flakes. The water-phase contact angle of oil-wet quartz flakes was measured by the YIKE-360A Contact Angle Analyzer;
(3) The oil-wet quartz flakes were soaked in HTLP-ME at different concentrations for 3 days. The water-phase contact angle of the quartz flakes mentioned above was measured as well.

In the wettability experiment, the deionized water droplet was added to the quartz flakes by the static drop method. The gas–water–solid three-phase contact angle was measured according to the three-point method. The average value was taken three times to obtain a more accurate three-phase contact angle.

### 2.4. Static Adsorption Experiment

Based on this property of the material, an ultraviolet spectrophotometer can be used to determine the absorbance of the substance, thereby deriving the change in its concentration or content. By measuring the relationship between concentration and absorbance of HTLP-ME, a standard curve of concentration and absorbance was made. The equation between absorbance and concentration was obtained in the linear fitting regression method.

The static adsorption experiment was carried out by the sealed oscillation equilibrium method. The pre-treated oil sands and different concentrations of HTLP-ME were placed in dry and clean bottles, with a solid–liquid ratio of 1:50, and then the bottles were sealed and placed in a rotary thermostatic oscillator, keeping the oscillation rate of 100 r/min and the oscillation time of 24 h to allow full absorption to achieve an adsorption equilibrium state. After the oscillation, the bottles were taken out. Separating the solid and liquid phases, the supernatant was sucked into the centrifuge tube with a dropper. The speed of the benchtop centrifuge was set at a high speed of 8000 r/min for 20 min. Subsequently, the supernatant was taken in the sample cell and the absorbance value of HTLP-ME after adsorption was determined by ultraviolet spectrophotometry. According to the standard curve equation, the concentration value of HTLP-ME after adsorption was obtained. As a result, the adsorption rate and the adsorption capacity of HTLP-ME at different concentrations were calculated.

The adsorption rate A is shown in Equation (1):

(1) $$A=\frac{C_{0}-C_{1}}{C_{0}}\times 100$$

where A is the adsorption rate, %; C_0_ is the concentration of HTLP-ME before adsorption, wt%; and C_1_ is the concentration of HTLP-ME after adsorption, wt%.

The adsorption capacity Γ is shown in Equation (2):

(2) $$\Gamma =\frac{C_{0}-C_{1}}{m}\times V$$

where Γ is the adsorption capacity, mg/g; C_0_ is the concentration of HTLP-ME before adsorption, wt%; C_1_ is the concentration of HTLP-ME after adsorption, wt%; V is the volume of HTLP-ME, mL; and m is the oil sand quality, g.

### 2.5. Emulsification and Solubilization

The kerosene and HTLP-ME were poured into the mixing cylinder with a stopper according to the volume ratio of 3:7. The emulsification experiment was carried out using the method of shaking bottles. The mixing cylinder with a stopper was turned upside down 10 times until the mixture was fully uniform [30]. After that, the mixture was instilled with a dropper and observed under an optical microscope to analyze the emulsification effect of HTLP-ME on the kerosene. HTLP-ME has a strong ability to increase the solubility of kerosene. After the kerosene and water phase were separated in the emulsification experiment, the lower aqueous phase was removed and centrifuged with a high centrifuged speed at 8000 r/min for 10 min. Then, the supernatant after centrifugation was taken and the changes in its microscopic morphology and droplet size were observed by TEM. According to the variation in the droplet size before and after solubilizing the kerosene, the droplet size growth coefficient, namely the solubilization coefficient (SC), was proposed for the first time. SC is the droplet size change rate of HTLP-ME, which represents HTLP-ME’s solubilization ability in kerosene. The larger the value of SC, the stronger the HTLP-ME’s solubilization ability to the kerosene. The droplet size growth coefficient SC is shown in Equation (3):

(3) $$\mathrm{SC}=\frac{d_{2}-d_{1}}{d_{1}}\times 100\%$$

where SC is the droplet size growth coefficient, %; d_1_ is the original droplet size of HTLP-ME, nm; and d_2_ is the droplet size of HTLP-ME after solubilizing the kerosene, nm.

### 2.6. Spontaneous Imbibition Distance Experiment

The CT scanning technology is used to visualize the distribution characteristics of the oil–water phase and the extension of the water saturation profile in the core. Therefore, the CT scanning technology can analyze the imbibition front and determine the imbibition distance [31]. The specific experimental processes are as follows:

(1) The CT values of air, kerosene, and imbibition agents were measured under the scanning voltage of 120 kV and scanning current of 160 mA;
(2) The dry core was placed in the core holder and the confining pressure was added to the holder until 12 MPa and it was then vacuumed. The CT scanning of the dry core was carried out to determine the CT values of each section of the core;
(3) Core treatment: The core surface was sealed with polytetrafluoroethylene (PTEE) material except the upper and lower ends of the core. The core was loaded into an intermediate container, which is saturated with kerosene by vacuuming and pressurizing at 12 MPa. The intermediate container was then aged in an oven for two weeks. After aging, the rock saturated with kerosene was taken out and the surface was wiped with oil rubbing papers. The rock was dried in the thermostat and weighed to calculate the mass difference (Δm) before and after being saturated with kerosene. One end face of the core was then sealed with PTFE material;
(4) The core saturated with kerosene was scanned again to determine the CT values of each section of the core;
(5) Spontaneous imbibition experiment: The spontaneous imbibition experiment was carried out at room temperature. The core sealed and saturated kerosene was placed into the Amott Cell. The imbibition agents were fresh water and HTLP-ME. It can ensure that only the reverse imbibition process occurred in the experiment. In the early stage of the experiment, the CT scanning was performed every 3 h and the initial position of each scanning was consistent. In the later stage of the experiment, the CT scanning can be performed twice a day;
(6) Data processing: According to the CT data of each section of the core, the imbibition front at different times was calculated and the reverse imbibition distance was determined;
(7) By recording the volume of cumulative discharged oil V_oi_ in the glass tube (precision 0.01 mL) at different imbibition times, the SIOR at that imbibition time was then calculated, as shown in Equation (4):

(4) $$\mathrm{SIOR}=\frac{V_{\mathrm{oi}}\rho }{\Delta m}\times 100\%$$

where i is the imbibition time, h; SIOR is the spontaneous imbibition oil recovery, %; V_oi_ is the cumulative oil volume at i time in the imbibition process, mL; ρ is the kerosene density, g/mL; and Δm is the core quality before and after saturated with kerosene, g.

## 3. Results and Discussions

### 3.1. Characteristics

#### 3.1.1. The Droplet Size and Morphology of HTLP-ME

**Figure 2:**
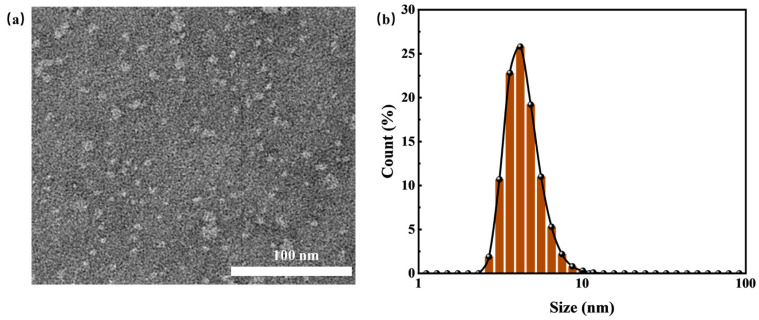
(**a**) The microscopic morphology; (**b**) The count of the size distribution of HTLP-ME.

Figure 2 shows the morphological characteristics of HTLP-ME under TEM observation and the droplet size by a particle size analyzer. As depicted in Figure 2, the “shell-core structure” of HTLP-ME has a uniform droplet size of 7.5 nm with no apparent agglomeration. The amphiphilic property of surfactants enables their distribution at the oil–water interface. Moreover, the synergistic effect of MOA15 and SS-2306 promotes the adsorption of surfactants at the oil–water interface, which significantly reduces the critical micelle concentration (CMC) [32]. The lower CMC value favors the formation of micelles, increasing their number while reducing their size. Additionally, the addition of co-surfactants reduces the rigidity and enhances the stability of surfactants’ adsorption layer at the oil–water interface. The synergistic effect of surfactants and co-surfactants is beneficial to the self-emulsification process of each component, which promotes the formation of numerous “shell-core structures” with a small droplet size in HTLP-ME.

#### 3.1.2. High-Temperature Resistant Experiment

The turbidity degree of HTLP-ME at different temperatures was determined using the turbidimetry method to quantitatively assess its temperature resistance of HTLP-ME. When a parallel light beam passes through the solution, part of it is absorbed and scattered while the remaining part is transmitted. By employing the measurement principle of 90° scattered light, the intensity of light scattered at a 90° angle to the incident light can be described by the Rayleigh formula. Within a specific range of turbidity and under constant incident light conditions, the intensity of scattered light is proportional to the solution’s turbidity. As a result, the Rayleigh formula can be expressed as Equation (5):

(5) IsI0= K′N

where I_s_ is the scattering light intensity; I_0_ is the incident light intensity; N is the number of particles per unit solution; and K′ is the coefficient.

Hence, the turbidity of the liquid sample is assessed based on the intensity of scattered light as it passes through the particles in the sample. A higher turbidity indicates lower solution clarity while a greater change in turbidity reflects a higher degree of solution instability.

**Figure 3:**
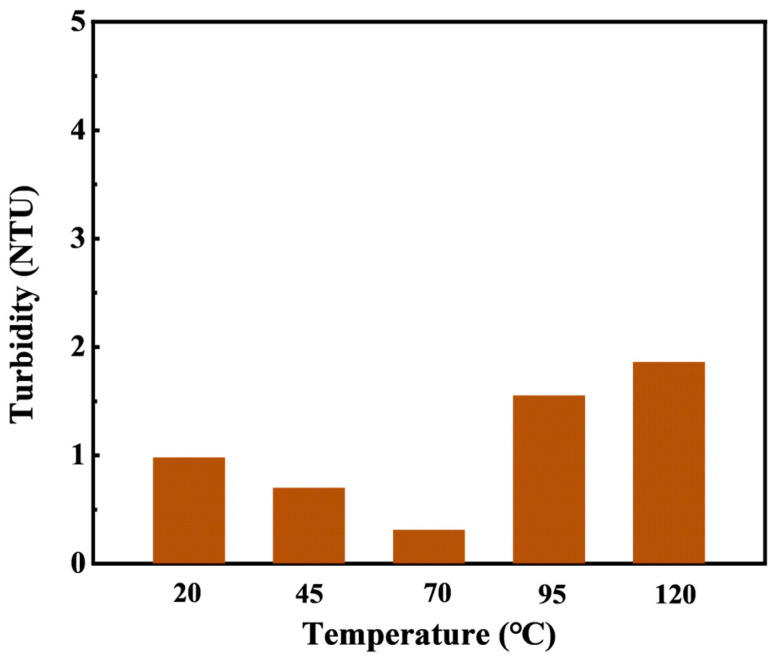
The turbidity of HTLP-ME variation with temperature.

The turbidity of HTLP-ME changes with temperature is shown in Figure 3. As the temperature increases, the turbidity of HTLP-ME decreases initially and then increases. The turbidity fluctuates in the range of 0~2 NTU with slight variation, indicating strong high-temperature stability. Cationic surfactant SS-2306 undergoes ionization in an aqueous solution and forms mixed micelles with nonionic surfactant MOA15. The hydrophobic carbon chains of the cationic surfactant penetrate and insert into the non-ionic surfactant micelles, leading to an increase in charge density and electrostatic repulsion of the outer layer of the micelles. This renders the mixed micelles difficult to aggregate. Additionally, the polar attraction of hydrophilic groups and the hydrophobic association of hydrophobic groups between nonionic and cationic surfactants inhibit the precipitation of nonionic surfactants at high temperatures. The synergistic effect of surfactants further enhances the turbidity point of the mixed surfactant system. Thus, the inclusion of SS-2306 elevates the turbidity point of the mixed surfactant system and significantly improves the temperature resistance of HTLP-ME.

### 3.2. Interfacial Properties

#### 3.2.1. Solid–Liquid Interfacial Property—Wettability

Wettability plays a crucial role in assessing the potential for spontaneous imbibition in tight cores. A common approach to characterize the core wettability is through measuring the surfaces’ contact angle. Hence, conducting the wettability experiments on HTLP-ME at different concentrations becomes necessary to elucidate the impact of wettability alteration capabilities on spontaneous imbibition.

**Figure 4:**
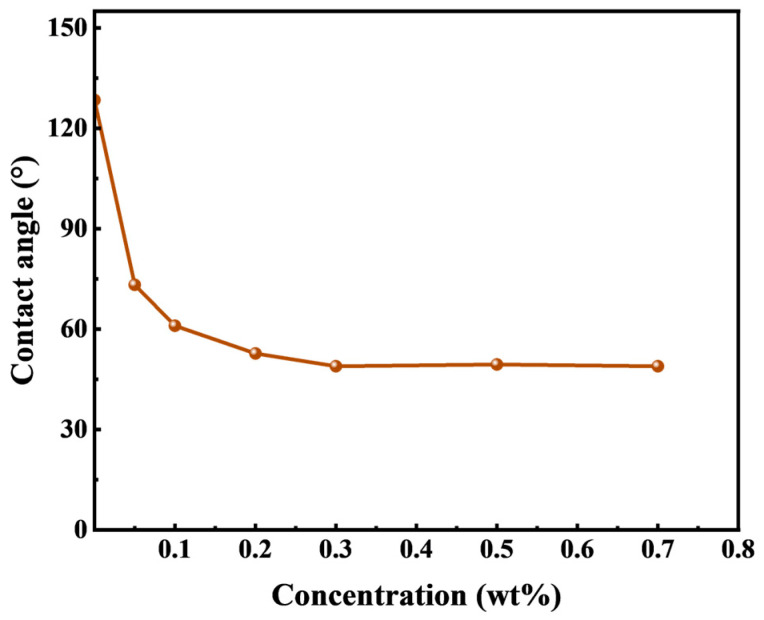
The variation in the water-phase contact angle on quartz flakes with concentrations of HTLP-ME.

Figure 4 illustrates the water phase contact angle of quartz flakes as a function of the concentration of HTLP-ME. The result indicates that initially, the water-wet quartz flakes can be transformed to an oil-wet state (about 130°) after immersion in silicone oil. When these oil-wet quartz flakes are subsequently immersed in different concentrations of HTLP-ME, the water phase contact angle gradually decreases, leading to a shift in wettability towards a water-wet state. This phenomenon occurs due to the adsorption of surfactants onto the surface of the quartz flakes [33,34]. Following the modification, the oil film is attached to the surface of the quartz flake and the surfactants present in the HTLP-ME can be absorbed onto the surface of the oil-wet quartz flake during soaking. The presence of the surfactant adsorption layer induces a transition from oil-wet to water-wet wettability. As the concentration of HTLP-ME increases, more surfactants are adsorbed onto the surface of the quartz flake, further enhancing the wettability alteration capacity and reducing the contact angle. At a concentration of 0.3 wt% of HTLP-ME, the adsorption of surfactants on the quartz flake’s surface reaches a saturation state. At this point, the wettability alteration capability is at its strongest, resulting in the smallest contact angle. Subsequently, as the concentration continues to increase, the contact angle remains constant, indicating that the wettability alteration ability becomes independent of the concentration.

#### 3.2.2. Oil–Water Interfacial Property—IFT

IFT plays a significant role in the spontaneous imbibition process of tight cores. In water-wet reservoirs, IFT is considered the driving force behind spontaneous imbibition. It facilitates the deformation of kerosene, diminishes the Jamin effect, and reduces migration resistance, thereby enhancing oil displacement efficiency. The IFT between HTLP-ME and kerosene is closely linked to the concentrations of HTLP-ME. Consequently, it is crucial to investigate the effects of HTLP-ME concentrations on IFT.

**Figure 5:**
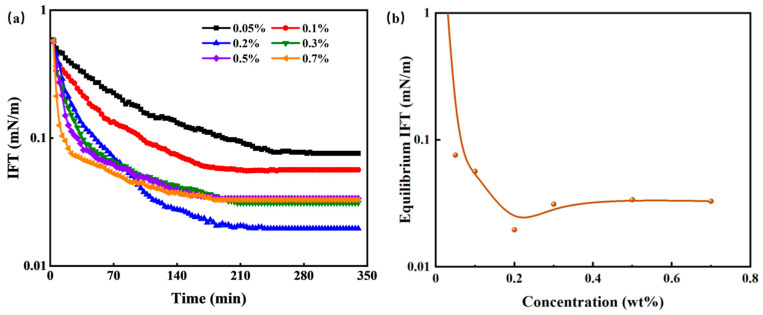
Effect of different HTLP-ME concentrations on the (**a**) dynamic IFT as a function of time and (**b**) the variation in equilibrium IFT.

Figure 5 presents the changes in dynamic IFT and equilibrium IFT between various concentrations of HTLP-ME and kerosene. In Figure 5a, the dynamic IFT of HTLP-ME at various concentrations exhibits a decreasing trend over time until it reaches a constant value. The rate of decrease in dynamic IFT is higher and the time required to reach equilibrium IFT is shorter with increasing concentrations of HTLP-ME. Figure 5b demonstrates that the equilibrium IFT initially decreases as the concentration of HTLP-ME increases. After that, it reaches the lowest value of 0.02 mN/m at a concentration of 0.2 wt% and then has a slight increase with the increase in concentration. The introduction of kerosene disrupts the “shell-core structure” and causes a redistribution of surfactants and co-surfactants at the oil–water interface [35]. Some surfactants and co-surfactants form the “shell-core structure” again, while others are adsorbed at the oil–water interface to reduce IFT. At lower concentrations, the surfactants and co-surfactants are predominantly adsorbed at the oil–water interface, resulting in a significant reduction in IFT. With increasing concentrations, the adsorption capacity of surfactants and co-surfactants at the oil–water interface increases, further decreasing IFT. At a concentration of 0.2 wt%, the surfactants and co-surfactants reach a saturated state of adsorption at the oil–water interface, resulting in the lowest IFT value. As the concentration continues to increase, the number of the “shell-core structures” increases. The formation of these structures requires amounts of surfactants, leading to competition with the surfactants adsorbed at the oil–water interface. Consequently, the number of surfactants adsorbed at the oil–water interface decreases, resulting in a slight increase in IFT. The IFT remains constant once a dynamic balance is achieved between the surfactants forming the “shell-core structure” and those adsorbed at the oil–water interface.

#### 3.2.3. The Adhesion Work Analysis

The core surface is more oleophilic and the migration resistance caused by its adsorption on the rock surface needs to be overcome in the process of kerosene migration. The migration resistance of kerosene is characterized by the adhesion work. Reducing the adhesion work between kerosene and rock surfaces can significantly decrease the migration resistance of kerosene, thereby improving the oil displacement efficiency through spontaneous imbibition. The relationship between the adhesion work, interfacial tension, and the contact angle is shown in Equation (6):

(6) $$W_{a}=\sigma_{\mathrm{ow}}\left(1-\cos\theta \right)$$

where W_a_ is the adhesion work between kerosene and the rock surface in the HTLP-ME oil–rock system, mN/m; σ_ow_ is the oil- HTLP-ME IFT, mN/m; and θ is the contact angle of water on the rock surface in the HTLP-ME oil–rock system,.

**Figure 6:**
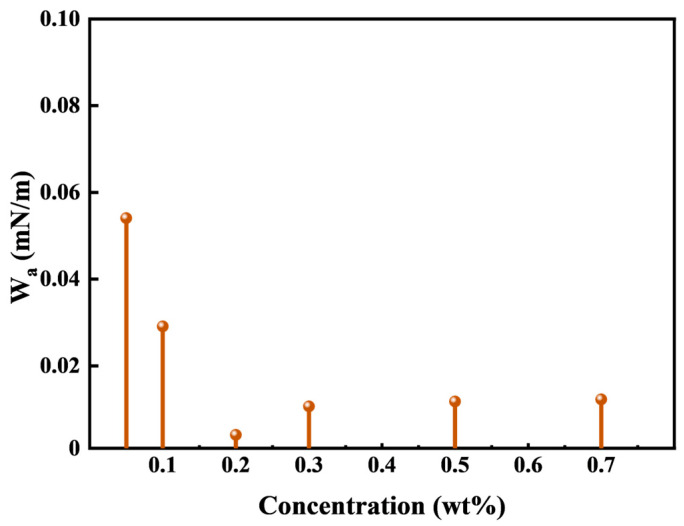
Effect of HTLP-ME at different concentrations on W_a_.

HTLP-ME is capable of reducing IFT at the oil–water interface and reversing the wettability of the rock surface from oil-wet to water-wet. Consequently, HTLP-ME can significantly reduce the adhesion work of kerosene. W_a_ characterizes the degree of reduction in IFT and the degree of change in wettability resulting from the addition of HTLP-ME. A smaller W_a_ indicates a stronger ability of HTLP-ME to reduce adhesion work, making it easier to strip kerosene from the rock surface. Consequently, a lower W_a_ value has a greater potential to enhance oil recovery. The variation in W_a_ with different concentrations of HTLP-ME is depicted in Figure 6. It can be observed that W_a_ initially decreases to the lowest value and then slightly increases before reaching a constant value as the concentration of HTLP-ME increases. When the concentration is 0.2 wt%, W_a_ reaches the minimum value of 0.004. At this point, the effect of reducing adhesion work is maximized, leading to enhanced oil deformation and the removal of the oil film from the rock surface. Consequently, the oil displacement efficiency is further improved.

### 3.3. Adsorption Experiment

The Lambert–Beer law, also known as Beer’s law, states that the absorbance value of a substance is directly proportional to its concentration within a specific wavelength range. The formula for the Lambert–Beer law is represented by Equation (7):

(7) $$A=\log\frac{I_{o}}{I}=\varepsilon \mathrm{lc}$$

where A is the absorbance of the sample; I_o_ is the intensity of incident light; I is the intensity of transmitted light when the incident light passes through the sample; ε is the proportional coefficient of light absorption; l is the optical path; and c is the concentration of the sample.

**Figure 7:**
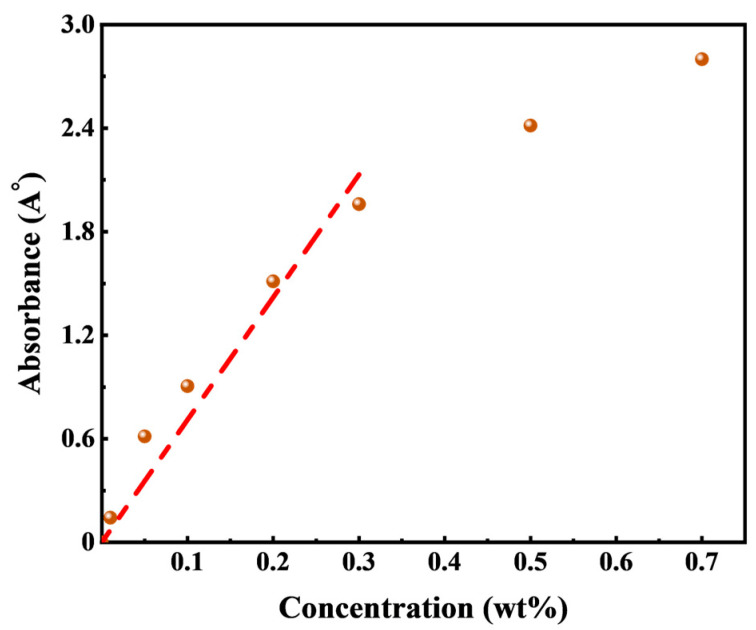
Standard absorbance–concentration curve of HTLP-ME.

To determine the characteristic wavelength of HTLP-ME, UV spectrophotometer measurements were conducted and it was found to be 198 nm. A standard absorbance–concentration curve was constructed by measuring the absorbance values of HTLP-ME at different concentrations at the characteristic wavelength of 198 nm, as shown in Figure 7. Within 0.3 wt%, the absorbance increases approximately linearly with increasing concentration; when exceeding 0.3 wt%, the absorbance shows a shift in which Beer’s law is not fulfilled.

When the concentration is in the range of 0.3 wt%, the standard curve of HTLP-ME is fitted using the linear regression method and then, the fitting equation is obtained as Equation (8). The solution is diluted in order to measure the absorbance when the concentration exceeds 0.3 wt%.

(8) y=9.0815x

**Figure 8:**
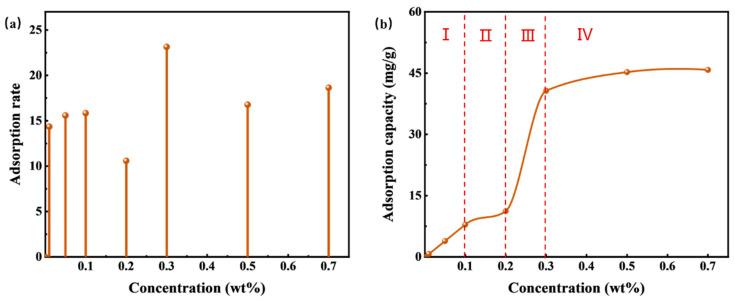
The static adsorption of HTLP-ME on the oil sand surface at different concentrations is about the (**a**) adsorption rate and (**b**) adsorption capacity.

The static adsorption experiment of HTLP-ME on the oil sand surface is conducted at different concentrations. Once equilibrium adsorption is reached, the absorbance value of HTLP-ME after adsorption is determined using a UV spectrophotometer. By utilizing the standard curve equation, the changes in HTLP-ME concentrations before and after adsorption are calculated. Relationship curves depicting the adsorption rate and adsorption capacity in relation to concentrations are plotted and presented in Figure 8. Figure 8a illustrates that the adsorption rate of HTLP-ME remains lower and fluctuates within the range of 10% to 25%, reaching its minimum value of 10.6% at 0.2 wt%. Initially, the surfactants in HTLP-ME exist in the form of a water phase shell. However, with the addition of oil sand during the static adsorption process, the surfactants undergo redistribution and disrupt the “shell-core structure”. While some surfactants continue to form the “shell-core structure”, others begin to be adsorbed onto the oil sand surface. The presence of the “shell-core structure” inhibits the adsorption of surfactants on the oil sand surface, resulting in a substantial decrease in the adsorption rate of HTLP-ME.

The curve in Figure 8b demonstrates that the static adsorption behavior of HTLP-ME on the oil sand surface aligns with the LS-type adsorption isotherm. The adsorption process can be roughly categorized into four zones:

(1) In zone I (c < 0.1 wt%), the surface of the oil sand carries a negative charge. At lower concentrations, physical adsorption occurs primarily due to the presence of the cationic surfactant SS-2306 which is influenced by electrostatic forces. This stage reveals ion-exchange adsorption where a single cationic surfactant molecule replaces a negative ion on the oil sand surface. Additionally, ion-pairing adsorption occurs when the cationic surfactant interacts with positively charged sites on the oil sand surface due to electrostatic interactions. During this stage, the surfactant adsorption has not yet reached saturation and the adsorption capacity increases linearly with concentration. The theoretical discussion of static adsorption at this stage is as follows.

The Stern-Grahame equation, shown in Equation (9), is applicable [36]:

(9) $$\Gamma =2\;r\;c\;\exp\;\left[\left(-z\;e\;\psi +\varphi \right)/\mathrm{RT}\right]$$

where Γ is the adsorption capacity of HTLP-ME, mg/g; r is the effective radius of the surfactant’s molecule; c is the concentration of HTLP-ME; z is the ion valence of the surfactant; e is the electronic charge; ψ is the Stern layer potential; φ is the adhesion work; R is the gas constant, 8.314 J/(mol·K); T is the absolute temperature. Taking the logarithm of Equation (9), Equation (10) is obtained:

(10) lnΓ=ln2r+lnc−z e ψRT+φRT

Equation (11) is obtained by a derivative of Equation (10) concerning lnc:

(11) $$\frac{d\;\ln\Gamma }{d\;\mathrm{lnc}}=1-\frac{\mathrm{ze}}{\mathrm{RT}}\cdot \frac{d\psi }{\mathrm{dlnc}}+\frac{1}{\mathrm{RT}}\cdot \frac{d\varphi }{d\ln c}$$

The adsorption process in Zone I is primarily driven by electrostatic forces. During this stage, the charge symbol and charge density of the Stern layer on the particle surface remain unchanged, resulting in a relatively constant electric potential. In theory, when φ = 0 and (dΨ)/lnc = 0 it follows that lnΓ/lnc = 1. In the curve of Zone I in Figure 8b, (d lnΓ)/(d lnc) is calculated using Equation (11) to be 1.04 which is close to the theoretical value of 1;

(2) In Zone II (0.1 wt% ≤ c < 0.2 wt%), as the concentration increases, the adsorption of cationic surfactant on the oil sand surface reaches saturation. Subsequently, there is slight adsorption of individual non-ionic surfactants on the surface, resulting in a modest increase in adsorption capacity. The main force governing adsorption in this stage is the van der Waals force;
(3) Moving to Zone III (0.2 wt% ≤ c < 0.4 wt%), with the continued increase in concentration, multiple adsorbed surfactant micelles form on the oil sand surface due to the hydrophobic association of the adsorbed surfactant molecules. The surfactants are rapidly and significantly adsorbed in the form of “semi-micelles,” leading to a rapid increase in adsorption capacity where (d lnΓ)/(d lnc) > 1. However, in the late stage of “semi-micelles” adsorption, the electrostatic repulsion between the adsorbed “semi-micelles” and the “shell-core structure” increases, resulting in a deceleration effect on the adsorption process. The adsorption capacity reaches its maximum value when this deceleration effect is strongest;
(4) In Zone VI (c ≥ 0.4 wt%), as the concentration further increases, a significant amount of “shell-core structure” forms in the solution. The “shell-core structure” exhibits strong electrostatic repulsion towards the adsorption layer. Additionally, the “shell-core structure” and the adsorbed surfactant micelles compete for surfactant molecules to maintain their respective dynamic equilibrium. Consequently, the adsorption capacity slightly declines after reaching its maximum value.

The results obtained from the interfacial performance experiments and static adsorption experiments provide evidence that HTLP-ME at a concentration of 0.2 wt% exhibits the lowest adhesion work reduction factor and the minimum adsorption rate. Therefore, it can be concluded that the strongest effect in reducing adhesion work and minimizing adsorption loss occurs at the concentration of 0.2 wt%.

Building upon the aforementioned experiments, further investigations are conducted using HTLP-ME at the optimal concentration of 0.2 wt%. These investigations include emulsification and solubilization of kerosene experiments as well as spontaneous imbibition experiments. Subsequently, the mechanisms by which HTLP-ME enhances the SIOR process are elucidated.

### 3.4. Emulsification and Solubilization Kerosene Experiments

**Figure 9:**
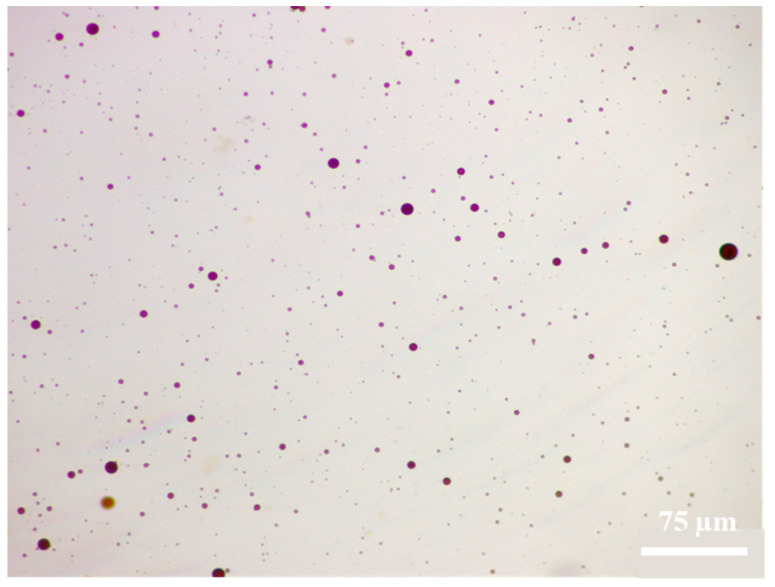
Morphology characteristics of the oil–water mixture under a 10× microscope.

The kerosene emulsification experiment of HTLP-ME is conducted using the shaking bottle method. The resulting oil/water emulsion is observed using an electron microscope and the microscopic morphology characteristics are depicted in Figure 9 under a 10× microscope.

In the experiment, when HTLP-ME contacts with kerosene, the continuous kerosene is broken up and emulsified, forming a single oil-in-water emulsion. The emulsion droplets are evenly dispersed within the water phase, exhibiting an average droplet size of only 2 μm. Upon contact with kerosene, the water phase shell of HTLP-ME is disrupted, leading to a strong molecular attraction between the kerosene and the lipophilic core in the system. The “shell-core structure” of HTLP-ME is consequently destroyed and then the lipophilic core and kerosene are brought together due to the similarity–intermiscibility theory between two oil phase components. This process reduces or even eliminates the molecular association effect among kerosene components, thereby breaking down the kerosene structure and forming the single oil-in-water emulsion. The presence of small-sized oil-in-water emulsion droplets reduces the likelihood of aggregation and recombination during migration. This phenomenon significantly weakens the Jamin effect, reduces flow resistance during displacement, and greatly enhances the migration speed and seepage capacity of kerosene.

**Figure 10:**
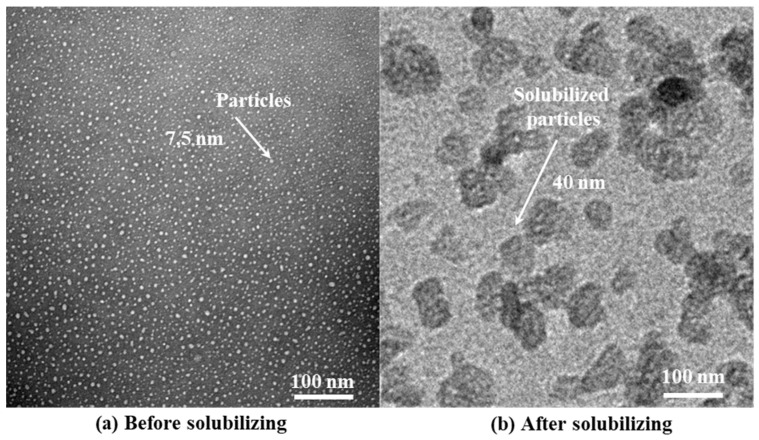
The changes in HTLP-ME’s droplet size (**a**) before and (**b**) after solubilizing the kerosene.

After the separation of oil and HTLP-ME, the aqueous phase is subjected to high-speed centrifugation at 8000 r/min. Following centrifugation, the droplet size distribution of HTLP-ME after solubilization is observed using transmission electron microscopy (TEM). Figure 10 displays the TEM image illustrating the droplet size before and after solubilizing kerosene. Prior to solubilization, the initial droplet size of HTLP-ME measures 7.5 nm. However, after solubilization, the droplet size increases to 40 nm, indicating a droplet size growth coefficient (SC) of 430%. This growth in droplet size is attributed to the redistribution and adsorption of surfactants as well as co-surfactants on the surface of the mixed oil phase, enabling the solubilization of a greater volume of the oil phase. During this process, a larger proportion of the kerosene can be displaced by spontaneous imbibition, leading to a significant increase in oil recovery. By solubilizing more oil phases, HTLP-ME facilitates the replacement of kerosene, thereby enhancing the overall oil recovery process.

**Figure 11:**
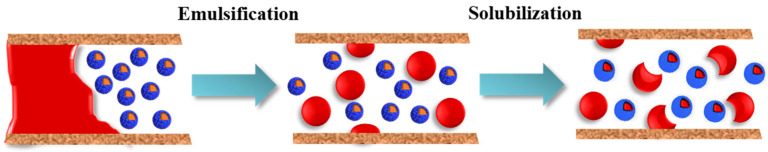
The schematic diagram of emulsification and solubilization of kerosene by HTLP-ME.

The schematic diagram of emulsification and solubilization of kerosene with HTLP-ME is shown in Figure 11. According to the Laplace equation, a pressure difference (∆P) is generated between two curved interfaces and always points towards the interior of the liquid, as shown in Equation (12). In the process of imbibition, there is a difference in droplet size between HTLP-ME “shell-core structure” and emulsified oil droplets, leading to the difference in ∆P of them. The droplet size of “shell-core structure” is smaller than that of emulsified oil droplets and, as a result, the ∆P is larger to produce a self-driving force (F_sd_) towards the emulsified oil droplets, as shown in Equation (13):

(12) $$\Delta P=\frac{2\sigma }{r}$$

(13) $$F_{\mathrm{sd}}=\Delta P_{1}-\Delta P_{2}=2\sigma \left(\frac{1}{r_{1}}+\frac{1}{r_{2}}\right)$$

where F_sd_ is the self-driving force, mN/m; σ is the IFT between HTLP-ME and kerosene, mN/m; r_1_ is the droplet size of the “shell-core structure” in HTLP-ME, nm; and r_2_ is the droplet size of the emulsified oil droplets, μm.

In the context of HTLP-ME, the F_sd_ plays a crucial role in solubilizing the emulsified oil droplets and displacing the kerosene during the spontaneous imbibition process. With the presence of F_sd_, the “shell-core structure” of HTLP-ME is capable of spontaneously solubilizing the emulsified oil droplets. Additionally, F_sd_ acts as a driving force, facilitating the displacement of kerosene during spontaneous imbibition, thus enhancing the SIOR process. In the specific case of HTLP-ME’s solubilization process, with parameters such as σ = 1.04 × 10^−4^ mN/m, r_1_ = 7.5 nm, and r_2_ = 2 μm, the calculated F_sd_ is approximately 2.76 × 10^4^ mN/m. It is worth noting that a higher F_sd_ during the solubilization process promotes the discharge of more kerosene by HTLP-ME. This increased F_sd_ enables HTLP-ME to effectively solubilize a larger volume of emulsified oil droplets and enhance the overall oil recovery process.

### 3.5. Spontaneous Imbibition Experiment

**Figure 12:**
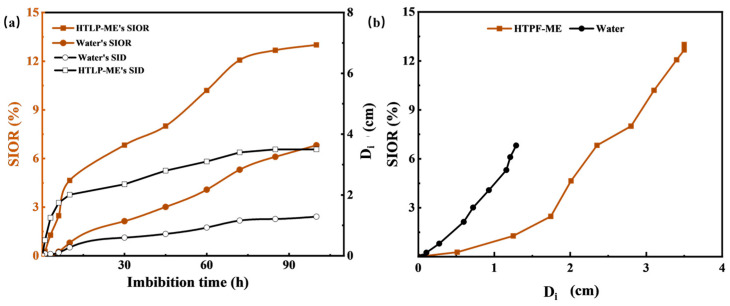
(**a**) The relationship of SIOR and Di with imbibition time; (**b**) The relationship between SIOR and Di.

The experimental results depicted in Figure 12 demonstrate the effects of reverse imbibition using fresh water and HTLP-ME on the spontaneous imbibition distance (D_i_) and SIOR in tight cores. In Figure 12a, as the reverse imbibition time increases, both fresh water and HTLP-ME gradually penetrate inward from the initial end of the core. The water saturation of the core noticeably increases, resulting in an extension of the reverse imbibition distance inward. Simultaneously, fresh water and HTLP-ME continue to displace kerosene through reverse imbibition, leading to an increase in SIOR. The rate of increase in D_i_ is significantly higher for HTLP-ME compared to fresh water. At the end of reverse imbibition, the imbibition front for HTLP-ME (3.51 cm) is much larger than that for fresh water (1.16 cm). This indicates that HTLP-ME has a more substantial displacement effect, penetrating deeper into the core. Similarly, with the progression of reverse imbibition time, SIOR for HTLP-ME experiences a sharp increase followed by a slower increase until it reaches the maximum value. The maximum SIOR achieved by HTLP-ME is 6.20% higher than that of fresh water, highlighting the significant effect of reverse imbibition using HTLP-ME. The curve depicting reverse imbibition oil recovery as a function of reverse imbibition distance (shown in Figure 12b) indicates that SIOR increases as the reverse imbibition distance increases. When the reverse imbibition distance reaches its maximum value, the SIOR also reaches its maximum value. Notably, both the reverse Di and SIOR of HTLP-ME are significantly higher than those of fresh water, further emphasizing the enhanced performance of HTLP-ME in the reverse imbibition process.

Figure 12 demonstrates that HTLP-ME not only increases the reverse imbibition distance but also enhances the oil recovery during reverse imbibition. This phenomenon can be attributed to the unique imbibition mechanisms exhibited by HTLP-ME.

Based on the exceptional performance and distinctive structural characteristics of HTLP-ME, the pressure difference between the two sides of the curved surface acts as the driving force behind spontaneous imbibition. Conversely, the adhesion work represents the resistance encountered during the displacement and migration of kerosene. The adsorption rate characterizes the effectiveness of HTLP-ME in displacing kerosene within porous media. Consequently, a lower adsorption rate is conducive to greater oil production, resulting in an inverse relationship with spontaneous imbibition. Furthermore, the droplet size growth coefficient serves as an indicator of HTLP-ME’s capacity to solubilize kerosene. A higher droplet size growth coefficient corresponds to a greater ability to promote oil production, thus establishing a direct proportionality with spontaneous imbibition. Based on the facilitation/inhibition of spontaneous imbibition by the above-mentioned characteristic coefficients, the spontaneous imbibition power factor P_w_ is defined as shown in Equation (14):

(14) $$P_{w}=(F_{\mathrm{sd}}-W_{a})\times \mathrm{SC}\times \left(1-\mathrm{AR}\right)$$

where P_w_ is the spontaneous imbibition power factor, mN/m; F_sd_ is the self-driving force, mN/m; W_a_ is the adhesion work of the HTLP-ME-kerosene-rock system, mN/m; SC is the droplet size growth coefficient; and AR is the adsorption rate.

The P_w_ serves as a crucial parameter for evaluating the effectiveness of HTLP-ME in spontaneous imbibition. A higher P_w_ indicates a more favorable oil displacement effect. HTLP-ME has an ability to reverse the wettability of rocks, transforming them into a water-wet state and promoting spontaneous imbibition in tight cores. Additionally, HTLP-ME significantly reduces the IFT between the oil and water phase, leading to a substantial reduction in the adhesion work of kerosene on rock surfaces. This reduction facilitates the emulsification and deformation of kerosene, minimizing migration resistance and greatly enhancing oil displacement efficiency through reverse imbibition.

Furthermore, HTLP-ME possesses a small-sized “shell-core structure” with a size of 7.5 nm. The presence of this structure helps inhibit the adsorption of surfactants on the rock surface, resulting in reduced adsorption losses with an adsorption rate of only 10.6%. Consequently, HTLP-ME can effectively penetrate the micro-scale and nano-scale pore throats of cores, extending the effective imbibition distance and increasing the sweep area.

Moreover, HTLP-ME emulsifies kerosene, forming small-sized oil-in-water emulsified oil droplets, and subsequently solubilizes these emulsified droplets. The pressure difference between the two sides of the curved interface serves as the driving force for kerosene displacement during spontaneous imbibition, significantly contributing to an increase in the spontaneous imbibition power factor. The spontaneous imbibition power factor plays a crucial role in characterizing the contributions of each factor in the spontaneous imbibition process. By quantitatively analyzing the spontaneous imbibition dynamic factor, the oil displacement mechanisms of HTLP-ME that greatly enhance SIOR can be revealed and studied. This approach provides a novel perspective for investigating the oil displacement mechanisms of microemulsion spontaneous imbibition, which is of utmost importance for the development of spontaneous imbibition in tight reservoirs.

## 4. Conclusions

(1) In conclusion, a novel high-temperature and low-phase microemulsion has been created for enhanced imbibition oil recovery and a thorough evaluation process has been established to identify the optimal concentration;
(2) The mechanisms of enhanced SIOR have been proposed to provide guidance for the spontaneous imbibition of tight reservoirs: The low interfacial tension and strong wettability alteration ability of the microemulsion reduce the adhesion work of kerosene, allowing for the deformation of kerosene and improved oil displacement efficiency in spontaneous imbibition; the “shell-core structure” with a small droplet size reduces adsorption loss and provides a self-driving force that can greatly extend the imbibition distance and enhance SIOR;
(3) HTLP-ME is expected to serve as an important technical support for the efficient development of tight reservoirs as a novel imbibition agent.

## Figures and Tables

**Table 1:** The parameters of the cores.

Number	Length/cm	Diameter/cm	Permeability/×10^−3^ µm^2^	Porosity/%
1	5.03	2.51	0.14	9.03
2	5.02	2.50	0.15	9.14

**Table 2:** Compositions and their mass ratio.

Quality of Each Component (g)	Nonionic Surfactant MOA15	Cationic Surfactant SS-2306	Oil Phase No.3 White Oil	Co-Surfactant Triethylene Glycol	Aqueous Phase Fresh Water
HTLP-ME	25	6	20	35	14

## Data Availability

The data presented in this study are available on request from the first author.

## References

[1] Zhao M., Song X., Lv W., Wu Y., Dai C. (2020). The preparation and spontaneous imbibition of carbon-based nanofluid for enhanced oil recovery in tight reservoirs. J. Mol. Liq..

[2] Raj I., Qu M., Xiao L., Hou J., Li Y., Liang T., Yang T., Zhao M. (2019). Ultralow concentration of molybdenum disulfide nanosheets for enhanced oil recovery. Fuel.

[3] Qu M., Liang T., Xiao L., Hou J., Qi P., Zhao Y., Song C., Li J. (2022). Mechanism study of spontaneous imbibition with lower-phase nano-emulsion in tight reservoirs. J. Pet. Sci. Eng..

[4] Zhou F.J., Su H., Liang X.Y. (2019). Integrated technology of high-efficiency fracture-mesh reconstruction and enhanced oil recovery in tight oil reservoirs. Petrol. Explor. Dev..

[5] Sun Y.P., Xin Y., Lyu F.T., Dai C.L. (2021). Experimental study on the mechanism of adsorption-improved imbibition in oil-wet tight sandstone by a nonionic surfactant for enhanced oil recovery. Pet. Sci..

[6] Xu D., Bai B., Wu H., Hou J., Meng Z., Sun R., Li Z., Lu Y., Kang W. (2019). Mechanisms of imbibition enhanced oil recovery in low permeability reservoirs: Effect of IFT reduction and wettability alteration. Fuel.

[7] Liang X., Zhou F., Liang T., Wang R., Su H., Yuan S. (2021). Mechanism of using liquid nanofluid to enhance oil recovery in tight oil reservoirs. J. Mol. Liq..

[8] Zhao H., Yang H., Kang X., Jiang H., Li M., Kang W., Sarsenbekuly B. (2020). Study on the types and formation mechanisms of residual oil after two surfactant imbibition. J. Pet. Sci. Eng..

[9] Karimaie H., Torsæter O., Esfahani M., Dadashpour M., Hashemi S. (2006). Experimental investigation of oil recovery during water imbibition. J. Pet. Sci. Eng..

[10] Xu G., Han Y., Jiang Y., Shi Y., Wang M., Zeng X. (2021). Reducing Residual Oil Saturation: Underlying Mechanism of Imbibition in Oil Recovery Enhancement of Tight Reservoir. SPE J..

[11] Arab D., Kantzas A., Torsæter O., Akarri S., Bryant S.L. (2021). A Crucial Role of the Applied Capillary Pressure in Drainage Displacement. SPE J..

[12] Kayali I.H., Liu S.H., Miller C.A. (2010). Microemulsions containing mixtures of propoxylated sulfates with slightly branched hydrocarbon chains and cationic surfactants with short hydrophobes or PO chains. Colloid. Surf. A.

[13] Bera A., Mandal A. (2015). Microemulsions: A novel approach to enhanced oil recovery: A review. J. Pet. Explor. Prod. Technol..

[14] Liu J., Sheng J.J., Wang X., Ge H., Yao E. (2019). Experimental study of wettability alteration and spontaneous imbibition in Chinese shale oil reservoirs using anionic and nonionic surfactants. J. Pet. Sci. Eng..

[15] Wang J.R., Yang S.L., Cao Y.J., Wang M., Yu J. (2020). Imbibition mechanism of tight oil cores and experiments of surfactants enhancing oil recovery. Sci. Techno. Eng..

[16] Strand S., Standnes D.C., Austad T. (2003). Spontaneous Imbibition of Aqueous Surfactant Solutions into Neutral to Oil-Wet Carbonate Cores: Effects of Brine Salinity and Composition. Energy Fuels.

[17] Paktinat J., Pinkhouse J., Williams C., Penny G.S. Microemulsion Reduces Adsorption and Emulsion Tendencies in Bradford and Speechley Sandstone Formations. Proceedings of the SPE International Symposium on Oilfield Chemistry.

[18] Santanna V.C., Curbelo F.D.S., Castro Dantas T.N., Neto A.D., Albuquerque H., Garnica A. (2009). Microemulsion flooding for enhanced oil recovery. J. Pet. Sci. Eng..

[19] Jeirani Z., Jan B.M., Ali B.S., See C.H., Saphanuchart W. (2014). Pre-prepared Microemulsion Flooding in Enhanced Oil Recovery: A Review. Petrol. Sci. Technol..

[20] Dantas T., Jsa P., Neto A., Neto E.L.B. (2014). Implementing New Microemulsion Systems in Wettability Inversion and Oil Recovery from Carbonate Reservoirs. Energy Fuels.

[21] Jeirani Z., Jan B.M., Ali B.S., Noor I., See C., Saphanuchart W. (2013). Formulation, optimization and application of triglyceride microemulsion in enhanced oil recovery. Ind. Crops Prod..

[22] Liu D., Xu J., Zhao H., Zhang X., Zhou H., Wu D., Liu Y., Yu P., Xu Z., Kang W. (2022). Nanoemulsions stabilized by anionic and non-ionic surfactants for enhanced oil recovery in ultra-low permeability reservoirs: Performance evaluation and mechanism study. Colloid. Surf. A.

[23] Nadeeka Upamali K.A., Liyanage P.J., Jang S.H., Shook E., Weerasooriya U.P., Pope G.A. (2018). New Surfactants and Cosolvents Increase Oil Recovery and Reduce Cost. SPE J..

[24] Yu F.W., Jiang H., Zhen FA N., Fei X.U., Hang S.U., Cheng B., Liu R., Li J. (2019). Characteristics and Imbibition mechanism of Winsor Ⅰ surfactant system in oil-wet porous media. Petrol. Explor. Dev..

[25] Su H., Zhou F., Liu Y., Gao Y., Cheng B., Dong R., Liang T., Li J. (2021). Pore-scale investigation on occurrence characteristics and conformance control mechanisms of emulsion in porous media. Petrol. Explor. Dev..

[26] Kumar N., Pal N., Mandal A. (2021). Nanoemulsion Flooding for Enhanced Oil Recovery: Theoretical Concepts, Numerical Simulation and History Match. J. Pet. Sci. Eng..

[27] Bui K., Akkutlu I.Y., Zelenev A., Saboowala H., Gillis J.R., Silas J.A. (2016). Insights into mobilization of shale oil by use of microemulsion. SPE J..

[28] Zhu T., Kang W., Yang H., Li Z., Zhou B., He Y., Wang J., Aidarova S., Sarsenbekuly B. (2021). Advances of microemulsion and its applications for improved oil recovery. Adv. Colloid Interface Sci..

[29] Solè I., Solans C., Maestro A., González C., Gutiérrez J. (2012). Study of nano-emulsion formation by dilution of microemulsions. J. Colloid. Interface Sci..

[30] Liu Z., Chai M., Chen X., Hejazi S.H., Li Y. (2021). Emulsification in a microfluidic flow-focusing device: Effect of the dispersed phase viscosity. Fuel.

[31] Yang Z., Liu X., Li H., Lei Q., Luo Y., Wang X. (2019). Analysis on the influencing factors of imbibition and the effect evaluation of imbibition in tight reservoirs. Petrol. Explor. Dev..

[32] Vale T., Roberta Magalhães R., Almeida P., Matos J.B.T.L., Chinalia F.A. (2020). The impact of alkyl polyglycoside surfactant on oil yields and its potential effect on the biogenic souring during enhanced oil recovery (EOR). Fuel.

[33] Qu M., Liang T., Hou J., Liu Z., Yang E., Liu X. (2022). Laboratory study and field application of amphiphilic molybdenum disulfide nanosheets for enhanced oil recovery. J. Pet. Sci. Eng..

[34] Wasan D.T., Nikolov A.D. (2003). Spreading of nanofluids on solids. Nature.

[35] Singh Y., Singh N.K., Sharma A., Singla A., Singh D., Rahim E.A. (2021). Effect of ZnO nanoparticles concentration as additives to the epoxidized Euphorbia Lathyris oil and their tribological characterization. Fuel.

[36] Hou J.R., Kang W.L. (1994). Petroleum carboxylate law of static adsorption experiment research on kaolin. J. Oilfield Chem..

